# Fatal Bacteremia Caused by *Campylobacter gracilis*, United States

**DOI:** 10.3201/eid2106.142043

**Published:** 2015-06

**Authors:** Takashi Shinha

**Affiliations:** Vanderbilt University Medical Center, Nashville, Tennessee, USA

**Keywords:** bacteremia, bacteria, Campylobacter gracilis, enteric infections, pneumonia, United States

**To the Editor:** *Campylobacter* species are well known to cause gastrointestinal infections in humans. However, extraintestinal illnesses caused by *Campylobacter* spp., including bacteremia, can also occur, primarily in immunocompromised persons (*1*). *Campylobacter gracilis* is a newly recognized species (*2*) that is commonly found in the oral flora and that has been associated with periodontal diseases and pleuropulmonary infections (*3*–*6*). Furthermore, a wide range of infectious etiologies caused by *C. gracilis* at different anatomic sites have been reported in the literature, suggesting its highly pathogenic potential (*7*,*8*). We describe a case of bacteremia due to *C. gracilis* complicated by pneumonia.

An 80-year-old man with a history of hypertension, hypertensive nephropathy, and　chronic obstructive pulmonary disease (COPD) was in his usual health status when he began having worsening productive cough, fevers, and malaise; he sought health care 5 days later at Long Island College Hospital (Brooklyn, NY, USA). A heavy smoker who was noncompliant with his COPD treatment, he had frequent episodes of COPD exacerbation necessitating chronic maintenance with oral steroid therapy.

At physical examination, the patient appeared chronically ill and had mild respiratory distress. His temperature was 100.8°F, blood pressure 124/67 mm Hg, pulse 106 beats/min, respiration 22 breaths/min, and oxygen saturation 94% on room air. His heart sounds revealed tachycardia without murmurs, and his lung sounds disclosed scattered wheezing and rhonchi.

Laboratory studies revealed a leukocyte count of 14,400 cells/mm^3^ (reference range 4,500–11,500) with 85% polymorphonuclear leukocytes, a hemoglobin level of 11.7 g/dL (reference range 14.0–18.0), and a platelet count of 174,000/mm^3^ (reference range 150,000–450,000). His sodium level was 133 mmol/L (reference range 135–145), potassium 4.6 mmol/L (reference range 3.5–4.5), bicarbonate 30 mEq/L (reference range 22–28), urea nitrogen 118 mg/dL (reference range 9–23), and creatinine 5.2 mg/dL (reference range 0.7–1.3). A chest radiograph showed consolidation with large pleural effusion in the right lung. 

He was empirically given vancomycin, cefepime, and azithromycin. Severe respiratory distress developed, and the patient died a few days later. Respiratory cultures at that time showed *Klebsiella pneumoniae* and *C. gracilis*. Blood cultures were positive for *C. gracilis*.

*C. gracilis*, originally known as *Bacteroides gracilis*, was transferred to the genus *Campylobacter* in 1995 after analysis of the cellular fatty acids, respiratory quinones, and proteins of *B. gracilis* and a comparison of them with the corresponding chemotaxonomic features of *Campylobacter* spp. (*2*). *C. gracilis* is a nonmotile, non–spore-forming, anaerobic gram-negative rod that uniquely requires formate and fumarate in its metabolism. *C. gracilis* primarily inhabits the gingival crevice and has been associated with a wide variety of periodontal diseases (*3*,*7*).

A study of 28 persons with chronic asymptomatic periradicular lesions showed *C. gracilis* in 6 (21.4%), including 2 (16.7%) of 12 who had acute apical periodontitis and 4 (23.5%) of 17 who had acute periradicular abscess (*4*). *C. gracilis* has also been isolated from other anatomic sites and has caused severe infections such as peritonitis, pneumonia, and bacteremia (*5*,*8*).

Our patient had *C. gracilis* bacteremia complicated by acute respiratory distress secondary to pneumonia. Although another gram-negative rod was isolated from the respiratory cultures, *C. gracilis* potentially played a major pathogenic role for this patient because of concomitant bacteremia that resulted in an unfavorable outcome. Pleuropulmonary infections with *C. gracilis* are not surprising because of the frequency of its detection in the human oral flora. In a study of 23 isolates of *C. gracilis* and their associated clinical diagnosis, 7 were from patients with lung abscess or empyema, and 2 were from those with aspiration pneumonia (*6*).

*Campylobacter* spp. are commonly associated with extraintestinal complications, including bacteremia, in immunocompromised hosts. In a study of 183 patients with *Campylobacter* bacteremia, the main underlying conditions were liver disease (39%) and cancer (38%). In that study, *C. fetus* was the most frequently identified species, found in 53% of the patients involved, followed by *C. jejuni*, *C. coli*, and *C. lari* (*1*). In another case report, *C. lari* bacteremia was described in a patient with multiple myeloma (*9*). Although uncommon, *C. gracilis* bacteremia has been reported in the literature (*8*). 

Optimal antimicrobial drug treatment for *C. gracilis* remains to be established. Available antimicrobial susceptibility patterns in the literature have shown conflicting results (*5*,*10*). In 1 study, penicillin susceptibility was 67% and cephalosporin susceptibility was 67%–84% in 23 isolates of *C. gracilis* (*6*).

Further research is warranted to elucidate the mechanisms of pathogenicity and virulence of *C. gracilis*. Its pathogenic potential should not be underestimated because of the spectrum of disease, severity of infection, and its possible high frequency of antimicrobial drug resistance. 
